# Fear of (re)injury and return to work following compensable injury: qualitative insights from key stakeholders in Victoria, Australia

**DOI:** 10.1186/s12889-017-4226-7

**Published:** 2017-04-11

**Authors:** Samantha Bunzli, Nabita Singh, Danielle Mazza, Alex Collie, Agnieszka Kosny, Rasa Ruseckaite, Bianca Brijnath

**Affiliations:** 1 The University of Melbourne, Department of Surgery, St Vincent’s Hospital, Melbourne, Australia; 2 Department of General Practice, School of Primary Care, Faculty of Medicine Nursing and Health Sciences, Monash University, Melbourne, Australia; 3 Department of Epidemiology and Preventive Medicine, School of Public Health and Preventive Medicine, Faculty of Medicine Nursing and Health Sciences, Monash University, Melbourne, Australia; 4 Institute for Work and Health, Toronto, Canada; 5 Transfusion Research Unit, Department of Epidemiology and Preventive Medicine, School of Public Health and Preventive Medicine, Faculty of Medicine Nursing and Health Sciences, Monash University, Melbourne, Australia; 6 School of Occupational Therapy and Social Work, Curtin University, Perth, Australia

**Keywords:** Return to work, Fear of (re)injury, Qualitative research

## Abstract

**Background:**

Return to work (RTW) is important for recovery post-injury. Fear of (re)injury is a strong predictor of delayed RTW, and therefore much attention has been given to addressing injured workers’ fear beliefs. However, RTW is a socially-negotiated process and it may be important to consider the wider social context of the injured worker, including the beliefs of the key people involved in their RTW journey.

**Methods:**

This paper involves data collected as part of a wider study in which semi-structured interviews explored RTW from the perspectives of 93 key stakeholders: injured workers, GPs, employers and insurance case managers in Victoria, Australia. Inductive analysis of interview transcripts identified fear of (re)injury as a salient theme across all stakeholder groups. This presented an opportunity to analyse how the wider social context of the injured worker may influence fear and avoidance behaviour. Two co-authors performed inductive analysis of the theme ‘fear of (re)injury’. Codes identified in the data were grouped into five categories. Between and within category analysis revealed three themes describing the contextual factors that may influence fear avoidance and RTW behaviour.

**Results:**

Theme one described how injured workers engaged in a process of weighing up the risk of (re)injury in the workplace against the perceived benefits of RTW. Theme two described how workplace factors could influence an injured workers’ perception of the risk of (re)injury in the workplace, including confidence that the source of the injury had been addressed, the availability and suitability of alternative duties. Theme three described other stakeholders’ reluctance to accept injured workers back at work because of the fear that they might reinjure themselves.

**Conclusions:**

Our findings illustrate the need for a contextualised perspective of fear avoidance and RTW behaviour that includes the beliefs of other important people surrounding the injured worker (e.g. employers, family members, GPs). Existing models of health behaviour such as The Health Beliefs Model may provide useful frameworks for interventions targeting the affective, cognitive, social, organisational and policy factors that can influence fear avoidance or facilitate RTW following injury.

## Background

A strong link exists between timely return to work (RTW) and injury recovery. Research has shown that a timely RTW following injury can raise an injured person's self-esteem, help maintain a sense of social standing and identity within the community, and accelerate physical and psychological recovery [1]. Timely RTW can also reduce the economic burden associated with health and legal expenses, staff retraining, and lost productivity [2]. In Australia, for example, the economic costs of occupational injuries are $60.6 billion or 4.8% of GDP per annum with the vast majority of this cost is (~74%) borne by the affected patient and their family [3]. The health costs of ‘worklessness’ are also substantial including a 20% increased risk of mortality and nearly thrice as much physical and mental morbidity compared to a non-injured person [4].

In light of this evidence, there have been growing efforts by compensable schemes in Australia, the UK, Canada and northern Europe to facilitate an injured person's RTW as safely and quickly as possible [5–7]. The key message is that one does not have to be completely fit to RTW and that injury recovery can continue following RTW [1]. The hurt versus harm logic underpins this message, i.e. that hurt – symptoms experienced during recovery – need not imply harm or damage, and that people may RTW despite experiencing some level of symptoms [8].

**Fig. 1:**
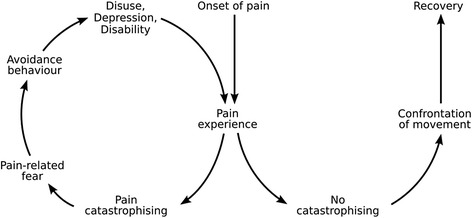
Fear Avoidance Model (Based on Vlaeyen and Linton 2000)

However, an injured workers’ fear of (re)injury in the workplace is one of the strongest predictors of delayed return to work (RTW) [9, 10]. According to the Fear Avoidance Model (see Fig. 1), widely applied in the rehabilitation literature, an injured worker’s belief that symptoms are a sign of harm or damage can sustain a cycle of fear and avoidance of activities of daily living, including work-related activities [11].

While the Fear Avoidance Model is a powerful tool for understanding an injured person's beliefs, it fails to place these beliefs within the social context of the injured worker, and does not consider how fear avoidance may influence other stakeholder’s willingness to facilitate the injured worker's RTW. Indeed, our research in the Australian context concurs with findings from other settings that RTW is a socially-negotiated process involving the injured worker in their unique social context, their employer, healthcare provider and insurance case manager [12–14]. It may be that the fear beliefs of other key stakeholders can influence an injured workers’ RTW. For example, a study by Linton et al. [15] described fear avoidance beliefs amongst healthcare providers that influenced their management of patients with back injuries. Almost one quarter of general practitioners (GPs) and physiotherapists in the study believed sick leave was a good treatment for back pain and two thirds advised patients that they should avoid painful activity [15]. Findings from other qualitative studies suggest that fear of (re)injury may be, at least in part, iatrogenic [16–18]. To date, how the beliefs of other non-medical stakeholders might influence an injured worker’s fear avoidance and RTW behaviour have not been explored. Experts have called for studies to decentralize the individual's perspective of injury and relocate it in a broader context of collective experiences [19]. Drawing on the perspectives of key stakeholders, in this paper we investigate the wider social context of the injured worker including the beliefs of key people in it, which may influence fear avoidance and RTW behaviour.

## Methods

### Study setting

This study was conducted in Victoria, the second most populated state in Australia, with a working population estimated at 2.8 million [20]. The relevant government regulator for work-related injury and illness in Victoria is WorkSafe Victoria. Employers are required to maintain workers compensation insurance. All work-related injuries or illnesses that exceed the threshold for healthcare expenses, or have required more than 10 days off work, must be lodged with WorkSafe Victoria via one of six private insurance companies. A medical certificate is required to lodge a claim and start the compensation payments for lost income, medical and other expenses. Limits on the duration of certificates exist; initial certificates can be for up to 14 days and subsequent certificates can be for up to 28 days. The GP plays a key role in certification, seeing 96% of all injured workers [21]. The estimated rate of medical certificates in general practice is 8.57 per 1000 workers, with musculoskeletal injuries accounting for approximately 40% of all certificates and mental health conditions for 10% [22].

### Research design

This study employed a qualitative description design [23]. We engaged four important stakeholder groups in the RTW process as identified in the literature and allowed them to speak about the RTW process, including factors that hinder and facilitate success.

### Sample and recruitment

Data were collected in metropolitan Melbourne between September and December 2012. The four stakeholder groups were recruited through different methods. An existing database of over 500 GPs who had consented to be contacted for research participation was used to recruit GPs. A fax was sent to each practice and GPs interested in participating contacted the research team. To be eligible, GPs had to have had experience within the past 12 months in treating injury compensation claimants. Efforts were made to purposively sample GPs by geographic practice location, gender, age and years of experience. Existing relationships between the research team and WorkSafe Victoria were used to identify compensation personnel. Invitations were mailed to agents, followed by a phone call 1 week later, with snowball techniques to identify additional agents. An existing database held by WorkSafe Victoria was used to identify employers and injured people. WorkSafe sent an initial letter to 200 employers and 200 injured people informing them they would receive a phone call from the research team and advising them they could opt-out if they did not wish to be contacted. Potential participants were then contacted 2 weeks later by the research team and invited to participate. WorkSafe Victoria were not informed of who eventually participated in the study and therefore anonymity was ensured. To be eligible, employers had to be over 18 years of age, come from medium to large business with 20 or more full time equivalent staff and more than one claim in the previous 12 months. This helped to ensure anonymity. Injured people were eligible where they were over 18 years of age and had a current claim related to musculoskeletal or psychological injury. We aimed to recruit 15–25 participants for each stakeholder group, consistent with previous qualitative studies in this area. Recruitment continued until patterns could be observed within and between the responses of each of the 4 stakeholder groups. The final sample included 93 participants: 17 injured persons, 25 employers, 25 GPs, and 26 insurance case managers.

### Data collection and analysis

Two research assistants experienced in semi-structured interview techniques conducted face to face interviews. Interviews lasted between 45 and 60 min. Open questions for all stakeholders included the questions: “Tell me about the process of recovery?”; “What do you think about RTW?”; “Can you think of what might facilitate RTW following injury?” and “Can you think of any barriers to RTW following injury?” Interviews were audio-recorded and transcribed verbatim on completion.

Two stages of data analysis were conducted. In the first stage, data was thematically analysed using inductive techniques [24, 25]. Analysis of early interviews occurred while data collection was ongoing. Four members of the research team met several times to discuss the emergent codes and themes and each transcript was coded and cross-checked. Differences of interpretation were resolved by consensus. In the final dataset, fear of (re)injury emerged as one of the salient themes across all 4 stakeholders groups. This presented us with a unique opportunity to analyse how the wider social context of the injured worker and the beliefs of key people surrounding them may influence fear and avoidance behaviour.

Transcripts were subsequently entered into NVivo10 (QSR International 2012, Melbourne) for further data analysis. In the second stage of data analysis, we performed inductive analysis of extracts in the theme ‘fear of (re)injury’ separately for each stakeholder group. This resulted in ten codes as listed in Table 1. These codes were grouped into categories that comprised of: 1. Perceived vulnerability, 2. Perceived severity, 3. Perceived benefits, 4. Perceived barriers and negative consequences and, 5. Stimulants to action. Data within and between each category were reviewed by two researchers and grouped into three themes. These three themes remained grounded in the participants’ experiences and described fear of (re)injury in the context of RTW following injury. The process of data reduction can be seen in Table 1.

**Table 1:** Process of data reduction

Codes	Categories	Themes
Consequences of (re)injury	Perceived severity	The risks versus benefits of return to work for the injured worker
Financial incentives	Perceived benefits	
Social responsibilities		
Stage in life		
Stress, depression, low mood	Perceived vulnerability	
Trust in employer	Perceived barriers	Workplace barriers to return to work
Availability of alternative duties		
Regulation of modified duties		
Willingness of employers	Stimulants to action	The beliefs of non-medical stakeholders
Liability concerns		

## Results

All injured workers had sustained their injury >3 months before; 71% had sustained their injury >9 months before. The injured workers were 71% male, with an average age of 48 years. The GPs were 72% male, with an average age of 52 years and 24 years experience as a GP. The employers were 36% male, with an average age of 45 years and 9 years experience in their current role. Insurance case managers were 15% male, with an average age of 34 years and an average of 7 years experience.

All participants recognised the importance of RTW, highlighting the economic and psychosocial benefits of work:

“The benefits of returning to work are many… they interact with their colleagues, they work, they get more money, they get happy, they seem to get better quickly” (GP#13, male (m), 67 years old (yo), 41years experience (ye))

Despite this recognition, less than half of all injured workers (*n* = 8 (47%)) had made an actual RTW. The majority (*n* = 12 (71%)) had been off work for more than 9 months; with one worker off work for 5 years. Analysis of their stories in conjunction with the experiences of GPs, employers and insurance case managers revealed three overarching themes describing the contextual factors that may influence fear of (re)injury and RTW behaviour 1. The risk of (re)injury versus benefits of RTW for the injured worker 2. Workplace barriers to RTW: addressing the source of injury and 3. Non-medical stakeholders’ fear of (re)injury beliefs. These barriers are elaborated below.

### The risk of (re)injury versus benefits of RTW for the injured worker

Instead of viewing the workplace as a healthy place which could promote recovery, most injured workers viewed it as a dangerous place where the risk of (re)injury was high:

“[I am] scared that the same thing’s going to happen. I am scared if I go to lift something or move something that my disc might slip. I don’t want to go through this whole process again” (Injured Worker #13, m, 20yo)*.*

Within this context of fear of (re)injury, injured workers weighed the benefits and risks of RTW. While a key motivation to remain off work was the injured workers’ belief that their injury needed protection and activities needed to be avoided until the symptoms had resolved, there were a host of other factors that appeared to influence whether they would RTW despite the perceived risk, or remain off work because of the risk. Such factors identified by participants in the study included how income was earned, age, having young children and family discord.

Some employers and insurance case managers believed that for injured workers who were paid on a fee-for-service basis such as a barber, or factory worker who were missing out on over-time during a busy period, the socio-economic benefits associated with RTW, i.e. the need to provide for one’s family, may have over-ridden the need to preserve one’s self. According to one employer:

“If we are not busy our absenteeism sky rockets… If it’s a hectic period where they are doing 20 hours a week overtime; it’s a lot of money [and] they will come back like that. They will take the initiative and, not force the doctor, but they will encourage the doctor to sign them back even if it’s on light duties” (Employer #3, m, 60yo, 10ye)

Age also influenced the motivation to RTW. According to employers, insurance case managers and GPs, older people who were approaching retirement had more financial incentives to remain off work than to return to the workplace. It may be that for older injured workers’ the desire to preserve one’s body over-rides the short-term need to earn money until retirement:

“I think some people when they are approaching retirement, you know 55 plus, I think some of them seriously start to think about workers compensation because they know they are only going to be here for another 5 years” (Employer #7, female (f), 47yo, 3ye)

“If they’re getting arthritis and they’re starting to get sore… they keep asking [for time off work], but it’s not going to go away. They are getting older” (GP#14, m, 53yo, 27ye)

For younger injured workers with dependent children, it was suggested that the financial and social incentives to remain off work may outweigh the perceived risk of RTW. According to one claims manager:

“A family member who has stopped work, is at home and now is able to look after the children, perhaps their motivation dwindles for return to work because they are at home and they are able to provide the childcare” (Insurance case manager #17, m, 38yo, 10ye)*.*

Family discord including marital breakdown, child maintenance arrangements and dealing with family illness were identified as factors that could also delay RTW for injured workers. But in such scenarios medical and non-medical factors were entangled as the stress, low mood and depression associated with family discord could increase negative thinking about the risks of RTW, reduce motivation to pursue the benefits of RTW, and amplify the pain experience. The GPs in the study emphasised the interplay between medical and non-medical factors that made the facilitation of RTW ‘always a bit complex’:

“If someone is going through a difficult marital breakdown or partnership breakdown or something of that nature, that can slow [RTW] down. But it doesn’t necessarily have to. If they’ve got child maintenance issues or whatever, they may be depressed. They may have sort of subclinical depression, and you know that they are miserable. But they insist that they’re just sore, it can be problematic. It’s always a bit complex” (GP#18, m, 60yo, 30ye)

### Workplace barriers: Addressing the source of injury

Workplace factors could also influence an injured workers’ perception of risk of (re)injury in the workplace, including confidence that the source of the injury had been addressed, the availability and suitability of alternative duties, and trust in the employer.

All groups of stakeholders recognised that the risk of (re)injury upon RTW could be decreased by removing or distancing the root cause of the injury. This was perceived as relatively straight forward in some cases, for example, the injured worker who has low back pain attributed to lifting, could RTW with restrictions on lifting. For individuals employed by large companies where alternative duties were readily available, the injured worker could be relocated to another department or a different job role where the risk of (re)injury was perceived to be lower. However, for individuals employed by small companies where alternative duties were not readily available, distancing the injured worker from the perceived source of injury was challenging:

“I can never go back to where I was because they won’t let me back in the warehouse because of my injury, and all of the admin computer type jobs are all upstairs and I can’t get upstairs” (Injured worker #5, f, 26yo)

“You might say “paperwork only” and the job might be landscape gardening. So there is no paperwork to do, and basically that becomes de facto ‘no duties’ because if there is no alternative duties then, there is nothing they can really do about that”. (GP#25, m, 50yo, 25ye)*.*

Similarly, removing or distancing from the source of injury could be difficult in certain situations such as bullying or harassment in the workplace when the perceived ‘cause’ of the mental health injury was the person the injured worker was being asked to go back and work with:

“It’s very difficult to get someone back to work when the reason they are away from work is the fact that they don’t want to work with the person they are being asked to go back to work with” (Insurance case manager #18, m, 61yo, 4ye)

“The difficulty is when it has been that stressful situation and bullying and stuff, sometimes then you don’t…there is no alternative if the environment was still, if the environment hasn’t changed” (GP#22, f, 49yo, 20ye)

In addition to the availability of alternative duties, the role that the employer played in ensuring that the risk of (re)injury remained low was recognised by the participants. A lack of trust between the injured worker and employer could raise doubts that the employer would act in the best interests of the injured worker and adhere to recommendations made by the GP:

“I was trying to return to work in the past, twice back last year. In November and December, and the things that made it hard was that my employer would say, ‘Yeah light duties, light duties.’ I’d go back to work for the first day or two it would be like light, and then on the third day it would be the same duties that I was doing. So then that would bring me back to square one” (Injured worker #13, m, 20yo).

### Non-medical stakeholders’ fear of (re)injury beliefs

Some employers and insurance case managers appeared reluctant to accept the injured workers back at work because of the fear that they might reinjure themselves. A poor understanding about the nature of the injury and a lack of awareness of the health benefits of work, appeared to make it difficult to accept that there may be a risk of (re)injury in the workplace. This may particularly be the case for complex injuries where a lack of clinical markers made it difficult to know how much to ‘push’ the injured worker:

“Sometimes the employer is in a position that they can’t return the person to work because they don’t know what they are safe to be doing. And the return to work co-ordinator – they may not understand the injury enough to know when that person should be going back to work” (Insurance case manager #21, f, 25yo, 3ye)

“You can get someone back to work too quick and then they reinjure themselves and that creates more problems down the track. Especially if the person is not in the same headspace as before. Now you have a mental health injury to deal with as well and that makes it a lot harder to overcome. You can’t tell straight away whether someone is going to have that reaction (Insurance case manager #12, m, 27yo, 3ye)

Legal and indemnity reasons for delaying RTW until the patient was 100% fit were also highlighted. Employers and insurance case managers were cognisant that at the end of the day, it was the employer’s legal obligation to keep the injured worker safe in the workplace:

“We have the lion’s share of obligations. We have the strongest role in return to work, the most to gain and the most to lose” (Employer #19, m, 39yo, 13ye)

“And sometimes [the employer] can’t have them back because if the client [injured worker] injures himself it falls on them” (Insurance case manager #13, f, 29yo, 12 ye)*.*

However, the existence of a Certificate of Capacity from a medical practitioner, legally obligates the employer to receive the injured worker back in the work place. For some employers, conflict was created between their legal obligation to receive the injured worker back in the workplace and their legal obligation to ensure that the injured worker did not reinjure themselves. This conflict appeared particularly salient when the employers doubted the validity of the Certificate of Capacity and didn’t feel they had the resources to manage the risk of (re)injury:

“Your hands are tied if you’ve got a Certificate of Capacity, even if you don’t believe it’s valid. There is not an awful lot anyone can do to challenge it and then you have the injured worker back and there is not a lot in the system in terms of resources to deal with them” (Employer #16, f, 38yo, 13ye)

## Discussion

Whilst fear of (re)injury is known to be an important predictor of RTW and is central to logic that governs RTW policy, until how little has been documented about the subjective experience of fear in the RTW context, nor how the fears of non-medical stakeholders may influence the RTW pathway. Findings from this large qualitative study offer important contributions to the fear avoidance model and RTW literature, extending current understandings of fear of (re)injury beyond the beliefs of the injured person and relocating it in a broader context of collective experience.

The first theme described in this paper is ‘the risk of (re)injury versus benefits of RTW for the injured worker’. We illustrated that whether an individual’s belief that they are at risk of (re)injury leads to fear and avoidance of work-related activities or not, may depend in part, on the value they place on competing goals. For example, “I need to get back to work to earn money for my family” may be juxtaposed against “If I go back to work and get hurt again how will I care for my family?” Affective states of the injured worker such as depression and low mood were identified as having a modulating effect on their perceptions of risk versus benefits associated with RTW. This is consistent with recent evidence that fear and avoidance are dynamic responses existing within the motivational context of the individual [26]. For example, an experimental study with healthy subjects showed that even when fear of injury is high, the frequency of fear avoidance behaviour can be reduced by the presence of a monetary reward [27]. This finding highlights the importance that health professionals understand the unique motivational context of each injured worker and tailor the hurt versus harm message accordingly, and that, at policy level, incentives are in place that emphasise the benefits of RTW.

The second theme described in this paper is ‘workplace barriers: addressing the source of injury’. We illustrate that whether an individual’s belief that they are at risk of (re)injury leads to fear and avoidance of work-related activities or not, may also depend on the extent to which they believe that measures taken to minimise risk will be effective. As highlighted by MacEachen et al. [8], early RTW can be counterintuitive to the injured workers’ understandings of recovery, and can amplify injury-related symptoms including pain and distress. Whilst fear avoidance is often construed as an irrational response on behalf of the worker, it may be a perfectly rational response if the worker believes that the workplace conditions leading to their injury have not changed (e.g. the pace of the work is the same, hazards have not been removed). In support of this stance, a prospective study of 1123 people with a workplace back injury found that one-fourth of workers reinjured their back following RTW and that fear avoidance beliefs at baseline predicted re-injury, thus raising the possibility that these workers were accurate in believing that their jobs would cause re-injury [28]. These findings highlight the need to ensure that processes are in place at work-sites so that the benefits of RTW do outweigh any risks and the injured worker remains in the workplace. This requires not only trust on behalf of the injured worker but also that the employer will provide appropriate alternative duties and a safe workplace by addressing the source of the injury. The capacity of the employer to do so is contingent on the size and nature of the business as well as their willingness to take action to remove the injury source (e.g. deal with the workplace bully, unsafe equipment). Unfortunately, a critical review of Australian compensation systems concluded that employers are rarely held accountable for failing to uphold their obligations to provide modified duties [29]. Policy may need to be changed and/or reinforced so that employers are held accountable to upholding the recommendations of capacity made by the healthcare provider. In the case that no alternative duties are available in the workplace, compensation bodies may need to play a more proactive role in placing the injured worker in an alternative workplace, ensuring they do not miss out on the health benefits of RTW.

The third theme described in this paper is ‘non-medical stakeholders’ fear of (re)injury beliefs’. Whilst hitherto the Fear Avoidance Model has been targeted at the level of the patient and clinicians without accounting for the beliefs of those in their greater social context [8, 30, 31], the findings of this study highlight that the fear of (re)injury beliefs of other, non-medical stakeholders in the RTW process may also be important in determining whether an injured worker returns to work or not. We emphasise the need to extend interventions beyond merely convincing injured workers that the benefits of work outweigh any risk, to include employers and compensation agents, in addition to healthcare providers. In particular, these findings highlight the importance of workplace interventions targeting employers own beliefs about activity and (re)injury, educating them about the health benefits of safe work and ensuring adherence to recommendations for light duties. Additionally, the importance of a trusting relationship between employers and the injured worker during the RTW journey needs further emphasis.

The key categories identified inductively in the data: perceived vulnerability, perceived severity, perceived benefits, perceived barriers and stimulants to action, are consistent with the key constructs described in a widely used model of the health behaviour, the Health Beliefs Model [32]. The Health Beliefs Model has been applied in the literature to understand behaviours in conditions characterised by avoidance and threat [33, 34]. According to the model, the adoption of a protective behaviour in response to a health threat will depend on an individual’s beliefs and attitudes towards the threat including: 1. Their perceived vulnerability to the health threat, 2. Perceived severity of the health threat, 3. Predicted benefits of a given health behaviour, 4. Perceived barriers and negative consequences of executing a health behaviour and 5. The events external to the individual that may stimulate them to conduct a given behaviour. This may include the somatic experience of disease symptoms e.g. pain intensity; beliefs about how others think a person should behave, and what a person has been told by key stakeholders. In this way, whilst the Health Beliefs Model is focussed at the level of the individual, it accounts for the beliefs of others in the social context that may influence the target behaviour.

**Fig. 2:**
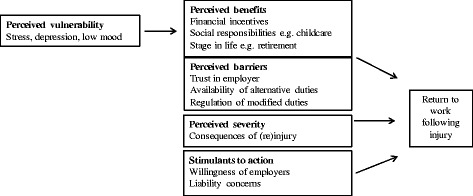
A Health Beliefs Model of return to work

Whilst to our knowledge, the Health Beliefs Model has not been applied in the context of RTW, we propose that the Health Beliefs Model may extend our understanding of fear of (re)injury in the context of RTW beyond the beliefs of the injured worker, to include their interactions with the environment around them. Viewed through the lens of the Health Beliefs Model, an injured workers’ RTW will be most likely when: 1. They perceive that remaining off work makes them more vulnerable to poorer health, 2. They perceive that the threat associated with (re)injury is sufficiently low 3. They believe that RTW will have benefits for their health, social and/or financial status 4. They have trust in their employer and can RTW in a modified capacity 5. Their employer is actively encouraging them to RTW (see Fig. 2).

While we have identified the Health Beliefs Model as being a theoretical lens through which we may consider RTW, it is acknowledged that other models of health behaviour can also account for the cognitive, social, organisational and systemic factors that may influence RTW. For example, Dunstan et al. [35] applied the Theory of Planned Behaviour [36] to understand the RTW expectations of injured workers and identified that the key determinants of expectations were the availability of modified duties, the social advantages of working, co-worker support, doctor’s advice and pain-related disability. Future research may explore the relative utility of the Health Beliefs Model in understanding fear of (re)injury in the RTW context versus other models of health behaviour that have been applied to understanding threat and avoidance behaviours such as the Common Sense Model and Protection Motivation Theory [37, 38].

There is now evidence that multidimensional interventions targeting identified barriers to RTW can be more effective at reducing fear avoidance and work absenteeism than single interventions educating individuals on the principles of hurt versus harm [30]. However, implementing multidimensional interventions can be challenging. Evidence suggests that employing health behaviour theories to understand the determinants of a given health behaviour can result in more effective, targeted interventions [39]. Interventions targeting Health Beliefs Model constructs have been effective in improving beliefs and attitudes towards participation in daily life activities following stroke [40], in diabetes [41] and coronary infarction [42]. Using the Health Beliefs Model as a framework may help direct multidimensional RTW interventions targeting an individuals fear of (re)injury, the beliefs of other stakeholders, and the organisational and political factors that can influence fear avoidance of the workplace or facilitate RTW. Future prospective studies are needed to explore if and how changes in Health Beliefs Model constructs may explain changes in fear of (re)injury and RTW outcomes.

To the best of our knowledge, this is the first study to include multiple stakeholder perspectives on RTW in the compensable injury setting. A limitation of our work is that it focuses on one jurisdiction, Victoria, Australia, and the findings may be of limited generalizability to other jurisdictions where different mandates control injury compensation. Further, we did not include all stakeholders in the RTW process, such as lawyers and allied health professionals. Selection bias may have also affected our findings as it is possible that individuals (particularly GPs and employers) with an interest in work injury claims agreed to participate. Finally, the data collection methods employed in this study mean that we are unable to assume data saturation was reached for the theme ‘fear of (re)injury’. However, the saliency of ‘fear of (re)injury’ across the transcripts of all stakeholders groups presented an opportunity to gain unique insight into how the wider social context of the injured worker and the beliefs of key people surrounding them, may influence fear and avoidance behaviour.

## Conclusions

In this study we have proposed that drawing on a well-established health behaviour theory can provide a contextual understanding of fear of (re)injury in the RTW setting. The findings highlight the importance that all stakeholders in the RTW process believe that the benefits of RTW outweigh any risks, and that processes are in place so that the benefits of RTW *do* outweigh any risks. To optimise RTW outcomes, theory-driven multidimensional interventions are needed to target the affective, cognitive, social, organisational and policy factors that may influence fear avoidance or facilitate RTW following compensable injury.
